# A Scoping Review on Wearable Devices for Environmental Monitoring and Their Application for Health and Wellness

**DOI:** 10.3390/s22165994

**Published:** 2022-08-11

**Authors:** Sara Bernasconi, Alessandra Angelucci, Andrea Aliverti

**Affiliations:** Dipartimento di Elettronica, Informazione e Bioingegneria, Politecnico di Milano, 20133 Milan, Italy

**Keywords:** wearables, air quality detection, exposure, pollution

## Abstract

This scoping review is focused on wearable devices for environmental monitoring. First, the main pollutants are presented, followed by sensing technologies that are used for the parameters of interest. Selected examples of wearables and portables are divided into commercially available and research-level projects. While many commercial products are in fact portable, there is an increasing interest in using a completely wearable technology. This allows us to correlate the pollution level to other personal information (performed activity, position, and respiratory parameters) and thus to estimate personal exposure to given pollutants. The fact that there are no univocal indices to estimate outdoor or indoor air quality is also an open problem. Finally, applications of wearables for environmental monitoring are discussed. Combining environmental monitoring with other devices would permit better choices of where to perform sports activities, especially in highly polluted areas, and provide detailed information on the living conditions of individuals.

## 1. Introduction

Air pollution is one of the major problems of the 21st century. It has been demonstrated that poor air quality has a strong impact on human health. In fact, as reported by He et al. [1], it causes a reduction in the average life expectancy of 1.8 years globally and 3 years in high-polluted areas of China. Nowadays, environmental monitoring is performed through fixed stations that collect levels of pollutants in its vicinity. For this reason, there is no complete monitoring of the territory, which is particularly important if we talk about air quality since there are rapid variations both from temporal and spatial points of view. That is why there is an increasing interest in developing portable and/or wearable systems that allow an accurate detection of air quality. This allows an increase in the awareness and help in the decision-making process, both from the personal and public points of view, leading to an improvement in air quality.

The present paper is a focused literature review of current, high-quality articles in the field of environmental monitoring. Information is obtained from scientific journals in various fields, such as biomedical, electronic, and environmental engineering. The articles were searched on Google Scholar, PubMed, Scopus, and Web of Science, using keywords such as “air quality”, “exposure”, “wearables”, “environmental sensors” or similar. It is organized as follows. In Section 2, pollutants and their impact on health are reported. Section 3 presents space and time resolution in pollution monitoring. Section 4 contains information about environmental sensors’ current technologies. In Section 5, commercial wearable and portable solutions, while in Section 6 wearables in scientific literature for environmental monitoring are reported, and Section 7 contains limitations in wearable detection. Then, in Section 8, exposure estimation adding activity recognition is explained and, in Section 9, air quality indexes are described. Finally, in Section 10, possible applications are highlighted.

## 2. Pollutants

Air pollution consists of the presence of a complex mixture of different chemical compounds in the air, which can be in the form of solid, liquid droplets, or gas. In general, an air pollutant can be defined as “any substance emitted into the air from an anthropogenic, biogenic, or geogenic source, that is either not part of the natural atmosphere or is present in higher concentrations than the natural atmosphere and may cause a short-term or long-term adverse effect” [2]. Air pollutants may have a natural, anthropogenic, or mixed origin, depending on their sources or the sources of their precursors. They can be divided into two classes: primary pollutants and secondary pollutants. Primary pollutants are directly emitted from the source and include particulate matter (PM), carbon monoxide (CO), nitrogen oxides (NO_x_), sulfur dioxide (SO_2_) and lead (Pb). Instead, secondary pollutants, for instance ozone (O_3_) or nitrogen dioxide (NO_2_), are the products of chemical reaction and microphysical processes and they are typically found far from the source [3]. The group of air pollutants that can potentially cause more damage to human health and to the environment is defined by the US Environmental Protection Agency (EPA) as “criteria pollutants” and include PM, CO, NO_2_, SO_2_, O_3_ and Pb. There are also many compounds that have been determined to be hazardous and are called air toxics, including as the main category volatile organic compounds (VOCs) [4]. Usually, for the outdoor environment, sulfur dioxide (SO_2_), oxides of nitrogen (NO, NO_2_, NO_x_ = NO + NO_2_), CO and O_3_ are considered. In the indoor environment, carbon dioxide (CO_2_), VOCs and, in some cases, also CO are the most present [5]. In Table 1, the major pollutants, their sources, and their impact on human health are reported.

### 2.1. Particulate Matter

**Figure 1:**
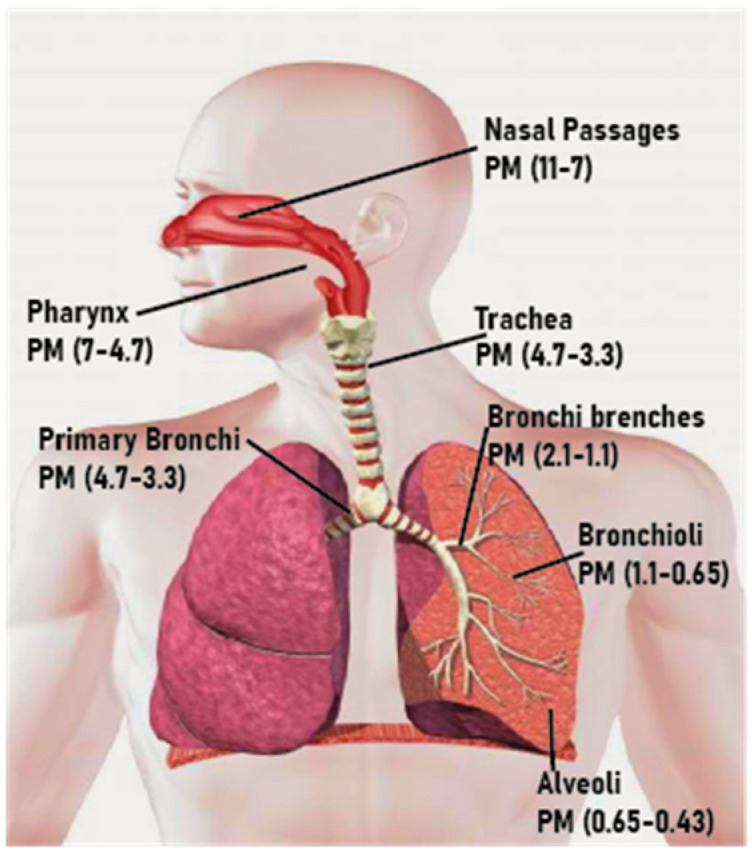
Deposition location of PM particles according to their diameter; adapted from [18].

The chemical constituents in PM are composed mostly by inorganic ions, such as sulfates, nitrates, and ammonium, but also by organic and elemental carbon, crustal material, particle-bound water, metals and polycyclic aromatic hydrocarbons (PAHs). PM is defined by the particle’s dimensions: the lower the diameter of the particle, the deeper through the respiratory system they can reach and so the more harmful for human health they are. Figure 1 shows where PM of different sizes is deposited. The two major subcategories are PM10 (with a diameter lower than 10 µm), which are also called coarse particles, and PM2.5 (with a diameter lower than 2.5 µm), called fine particles [10]. PM10 and PM2.5 are the most widely studied air pollutants because PM poses one of the greatest risks to human health [11].

The sources of coarse particles are mostly due to resuspension of soil tracked onto streets or of industrial dust. Sources of fine particles are the combustion of coal, oil and gasoline [12], and diesel engines account for the generation of 90% of fine particles in cities. In 2005, a study conducted in a classroom during a week [13] demonstrated that an important portion of PM10 comes from within the room, even if outdoor pollution has an impact on it. Therefore, humans have an impact on PM generation.

Inhaling PM can cause premature death if heart or lung diseases are present as well as nonfatal heart attacks and irregular heartbeat [14]. Moreover, it aggravates asthma, decreases lung function and increases respiratory symptoms, such as irritation of the airways, coughing and difficulty breathing [15]. PM2.5 has also been shown to have an impact on the development of hypertension [7]. However, as highlighted by Zhao and Kettsu [16], the effects of PM do not only influence physical health but also mental health. In fact, recent studies [8,17] found a connection between high levels of PM and depressive symptoms. An interesting point of view is the one proposed by He et al. [1], which focuses on the labor productivity reduction connected to exposure to PM2.5. Exposure affects productivity in terms of the physical functioning of the human body and cognitive functions of the human brain.

### 2.2. Nitrogen Dioxide

NO_2_ are secondary contaminants mainly because they are the product of a photochemical reaction of NO with O_3_. During daytime hours, NO_2_ is converted back into NO with the consequent regeneration of O_3_ [19]. As stated by EPA, road traffic is the principal outdoor source of nitrogen dioxide. The most important indoor sources include tobacco smoke and gas-, wood-, oil-, kerosene- and coal-burning appliances.

Evidence has been found that NO_2_, among other traffic-related pollutants, has a causal role in mortality and in the development of chronic respiratory diseases. There is an ongoing debate on whether NO_2_ is directly responsible for health effects or is only an indicator of other pollutants, including particulate matter and specific constituents such as metals, polycyclic aromatic hydrocarbons and other organic matter [10]. Some epidemiological studies suggest associations of long-term NO_2_ exposures with respiratory and cardiovascular mortality and with children’s respiratory symptoms and lung function that are independent of particulate matter mass metrics. Such studies also suggest that NO_2_ aggravates respiratory symptoms, especially for vulnerable categories, such as children, older adults and asthmatics [20].

### 2.3. Ozone

Ozone is a strong oxidant agent with a variety of effects, including lung inflammation, alveolar epithelial damage and changes in the chemical composition of lung lavage fluids [21]. Tropospheric ozone is the second most important atmospheric pollutant after particulate matter with respect to its impact on human health. A study [19] demonstrated that ozone concentration increases with increasing solar radiation and temperature and that both the NO and NO_2_ decrease correlate with an increase in O_3_. This relationship can be used to forecast O_3_ values.

Epidemiological studies showed that an exposure in the range of 160–360 μg/m^3^ O_3_ for a duration of 1–8 h may reduce lung function. Ground level ozone can inflame the lining of the lungs and, if the exposure is prolonged, it can permanently damage lung tissue [10]. Both NO_2_ and O_3_ exposure have been demonstrated to trigger an inflammatory response, including in vitro and in vivo increase in IL-8 concentrations [22]. There has been a study in Iran that showed that high O_3_ concentrations and/or an increased number of days on which people are exposed to this pollutant likely result in adverse health outcomes. The result of this study was in agreement with previous studies [23].

### 2.4. Carbon Monoxide

Carbon monoxide is a colorless, non-irritant, odorless, and tasteless toxic gas. It is produced by the incomplete combustion of carbonaceous fuels, such as wood, petrol, coal, natural gas, and kerosene. CO can be released when engines burn fossil fuels, or it can be emitted from vehicles, furnaces, and heaters. Carbon monoxide is produced indoors by various combustion sources (cooking and heating).

It has also been assessed that CO concentration increases in indoor environments where there is no ambient ventilation after smoking. In fact, CO is present in mainstream smoke, which is directly inhaled by active smokers at levels from 5 to 22 mg/cigarette while, in side-stream smoke, from 9 to 35 mg/cigarette. Side-stream tobacco smoke and the smoke exhaled by active smokers are the most important components of second-hand smoke (SHS, also called environmental tobacco smoke (ETS)) [24].

In the human body, it reacts with the hemoglobin to form carboxyhemoglobin: reversible binding occurs on the same heme site where oxygen binds, thus preventing the transport of oxygen to the tissues. The reduction in the amount of oxygen reaching the body’s organs and tissues can cause a sensation of dizziness, fatigue, and headache as minor symptoms, while it can be very dangerous for people with heart diseases. There are specific guidelines for indoor carbon monoxide: excursions above 35 mg/m^3^ should not occur more than once per day and can be very dangerous if they happen while the person is performing light exercise or where people affected by respiratory or cardiovascular diseases live.

### 2.5. Sulfur Dioxide

Sulfur dioxide is a primary pollutant, mostly produced by the combustion of coal or oil, but it can be also produced by factories pertaining to chemicals, paper or fuel [25].

SO_2_ has a great impact on human health, causing irritation of the membranes of the nose, throat and lung [26]. In fact, it reacts with the moisture present in the nose, nasal cavity and throat, leading to nerve damages [27]. The provoked irritation and inflammation of the respiratory system can lead to cough and difficulty in taking deep breaths. In 2017, SO_2_ was added in the list of carcinogens by WHO causing lung cancer. It also be related to obesity and heart disease [28].

Sensitive groups are people with lung diseases, children, and older adults. However, healthy people can also experience respiratory symptoms when exercising, starting at 1.6 ppm. An exposure of few minutes at 8–12 ppm causes irritation to the throat and at 20 ppm cough and eye irritation immediately appear.

### 2.6. Volatile Organic Compounds

A volatile organic compound is defined by EPA as any compound of carbon, excluding carbon monoxide, carbon dioxide, carbonic acid, metallic carbides or carbonates and ammonium carbonate, that participates in atmospheric photochemical reactions. In fact, VOCs are organic chemicals that, when released into the atmosphere, can react with sunlight and nitrogen oxides (NO_x_) to form tropospheric ozone [29]. VOCs can be either primary or secondary pollutants. 

Humanmade VOCs are mainly produced by the petrochemical industry, technological processes, and the combustion of conventional fuels. Internal sources are derived from human activities, such as cooking or smoking and from materials from which a building is made. Additionally, floor surface materials (PVC/vinyl and linoleum) are potential sources, as highlighted by a study in primary schools in Porto [9]. A considerable number of VOCs are generated by cells or microorganisms and can be detected in different biological specimens, such as exhaled breath [30].

Most of the VOCs in the indoor environment are at relatively low concentrations and a few of them (e.g., benzene and formaldehyde) are highly toxic; in general, they cause sensitivity and respiratory irritation [31].

### 2.7. Carbon Dioxide

Carbon dioxide is a colorless, tasteless, odorless and non-flammable gas that is heavier than air and may accumulate in lower spaces, causing a deficiency of oxygen.

CO_2_ is a primary pollutant. It is naturally present in the Earth’s atmosphere as a trace gas and can be produced both from natural sources, for instance, by human expiration, and human sources, such as deforestation and the burning of fossil fuels such as coal, oil, and natural gas.

According to the World Meteorological Organization, the globally averaged CO_2_ concentration in the atmosphere reached 400 ppm for the first time in 2015 and it is currently increasing by 2 ppm a year.

The main source of CO_2_ in the non-industrial indoor environment is human metabolism. Higher concentrations of CO_2_ in the indoor environment cause a broad range of diseases and symptoms. Recent studies have reported a linear increase in pCO_2_ (partial pressure of carbon dioxide) in the blood at CO_2_ exposures in the range from 500–600 to 4000–5000 ppm. A concentration lower than 5000 ppm is considered safe for eight-hour exposure and it does not immediately affect health. However, a concentration above 1000 ppm affects cognitive function and comfort [32]. Symptoms worsen up to unbearable dyspnea, vomiting, disorientation, hypertension and loss of consciousness for concentrations above 100,000 ppm [33]. Moreover, a study [34] that assessed air quality and thermal comfort in the workplace highlighted the correlation between CO_2_ levels and sick building syndrome (SBS) symptoms, which are all the heath symptoms caused by poor indoor air quality, as stated by WHO. Specifically, SBS symptoms are related to the upper and lower respiratory tract, eyes and skin and include headache, fatigue, and difficulty in concentrating.

Another parameter of interest is the partial pressure of transcutaneous CO_2_ (PtCO_2_), which measures the CO_2_ coming out of the skin and approximates the partial pressure of arterial CO_2_ (PaCO_2_), i.e., the level of CO_2_ in the blood [35]. Environmental CO_2_ can influence the levels of CO_2_ in the blood, together with pathological conditions.

## 3. Space–Time Resolution in Pollution Monitoring

In general, when the estimation of the pollution exposure of humans is to be studied to assess the health impact of different gas or particles, data are taken from central site (CS) monitors. A brief clarification is necessary: there is a difference between air pollution and exposure, which is the quantity of pollutant that an individual encounters.

According to Idrees and Zheng [36], air pollution monitoring systems can be classified according to location and surrounding environment in outdoor, indoor and industrial areas. These three categories determine the characteristics and limitations of the air pollution monitoring system. We analyzed outdoor and indoor environments.

Regarding the outdoor environment, different studies highlighted the strong spatial variability of air pollution over a short distance (<100 m) [37], suggesting a paradigm shift from constant air quality characterization to a variable in time and space characterization. In fact, air pollution is strongly dependent on pollution sources and air flow, which is also difficult to predict with a sophisticated numerical modeling [38]. For instance, in an urban area where high buildings are present on both sides of a street, air can be strongly polluted because low air dispersion is allowed and, thus, high local pollution can be detected, but if the nearest fixed station is not in this “street canyon”, data extracted about pollution cannot be representative of the situation in that street. Moreover, as reported by Arano et al. [11], the time frequency resolution of the available data is also a known limit in exploiting data coming from a fixed station.

Another side of the spatial resolution problem is the evaluation of indoor pollution.

According to the considered study, between 80% and 90% of the time in our daily routine takes place in closed indoor environment, for instance houses, workplaces, public or private transportation, gyms, and other similar environments. Indoor air pollution is a leading cause of 1.6 million premature deaths according to WHO [39]. In addition, strong evidence in the literature shows that poor indoor air quality can also affect the learning and cognitive ability and the productivity of humans [33,40]. This is the reason why considering only indoor air quality to estimate exposure can be considered better than using data coming from outdoor fixed stations only.

A recent study [41] assessed exposure to PM2.5 with two different approaches: in the first approach, it considered exposure estimation counting only data of residential-based air pollution, and the second one also considered exposure in workspaces and all activity locations. The first approach proved to underestimate the exposure of a 13%, but much of the error is controlled by considering workspaces. Nevertheless, this estimation bias increased considerably for workers who must travel along commuting distances, reaching a 61% underestimation if the commute distance is more than 30 km. Therefore, considering only indoor air quality can lead to errors in the estimation of exposure.

A possible way to cope with this issue is to evaluate the ratio between indoor/outdoor pollution and then using outdoor data to evaluate indoor pollution, adding the contribution of sources or ventilation [38]. This means that time–activity diaries, Artificial Neural Networks [42] or GPS are necessary to retrieve the trajectories of an individual. However, even if this approach seems to be more accurate, errors caused by a lack in spatial resolution are still present.

All these factors suggest that, if we want actual data of air pollution to evaluate exposure, a portable or even better wearable solution is needed [43].

## 4. Environmental Sensors

From the sensors side, most of the small commercial sensors available belong to four different technologies: resistive sensors, electrochemical (EC) sensors, dispersive infrared radiation absorption (NDIR) sensors and photo-ionization detector (PID) sensors. Sensors for environmental applications can be divided, as reported by Narayana et al. [44], into two categories: gas sensors (GS) and particulate matter sensors (PMS). Before describing these sensors in detail, it can be useful to the reader to have some definitions. Sensitivity is defined as the relationship between input and output variations. In the context of this review, a high sensitivity sensor is a sensor in which small variation in the concentration of a pollutant corresponds to a great variation in the output electrical signal.

Selectivity determines whether a sensor can respond selectively to a group of analytes or even specifically to a single analyte. A detailed description of different sensors is now presented and, at the end of Section 4, a summary table with their advantages and disadvantages is reported.

### 4.1. Gas Sensors

#### 4.1.1. Metal Oxide Semiconductor Sensors

Metal oxide semiconductor (MOS) sensors are used to detect gases such as nonmethane hydrocarbons (NMHCs), CO, CO_2_, NO, NO_2_, NO_x_ and O_3_ [45]. Resistive metal oxide gas sensors (tin oxide) are one of the most used types of sensors in air quality monitoring. Tin oxide, which is the sensing element, is chosen because of its high variation of resistivity and the wide range of gases with which it reacts. Resistive metal oxide gas sensors are also the most widely used because of their low price, size, and consumption.

In clean air, O_2_ is adsorbed on the metal oxide layer. Due to its high electrical affinity, oxygen takes free electrons in metal oxide and bonds to them. The potential barrier is so high that no current can flow; so, in clean air, the resistance is approximately infinite.

In the presence of polluted air, the pollutant bonds with oxygen molecules, which leaves free electrons in the metal oxide so that the potential barrier is lower and the current can flow. The amount of current is inversely proportional to the resistivity of the material, which, in turn, depends on the concentration of the pollutant. The chemical reaction of gases and adsorbed oxygen on the tin dioxide surface varies depending on the reactivity of sensing materials and working temperature of the sensor. To increase the rate of the reaction, the sensing element is warmed up to 300–500 °C by a heater, always encapsulated in the device [46]. In the datasheets, the appropriate conditioning circuit is suggested. In fact, according to the specific gas, different values of resistance need to be placed in series with the power supply to guarantee the right temperatures on the heaters. An operational amplifier can be used to obtain a better quality of the output signal.

As the conductance of MOS materials is proportional to the number of free electrons: the more reductive the gas is, the more free electrons there will be available and thus the more conductive the materials will become. Resistive metal oxide sensors have a high sensitivity and an input range that spans from a few ppb to several thousand of ppm of gas, wider than other type of gas sensors. This characteristic reduces system design and maintenance when there are different ranges to monitor [47]. However, the sensitivity of the MOS sensor is influenced by other gases and atmospheric conditions. MOS sensor is sensitive to almost every volatile compound (reducing or oxidizing) and toxic gases such as NO_x_ or VOCs. Usually, there are tables of equivalent responses in the datasheet to manage this cross-sensitivity. To calibrate the sensor, temperature and humidity need to be accurately measured to model their effect on the sensor. However, MOS sensors have a high resilience against environmental conditions and great longevity (almost 10 years). The response time is of few minutes, which is considered short in the field of environmental sensing.

Stability is the major problem. In fact, the sensing element decreases its conductance over time, changing the sensors’ accuracy. Therefore, MOS sensors need to be recalibrated. Different studies proposed a wide range of methods to model the response, from parametric to non-parametric models, using neural networks, for example.

The presence of an electric heater requires considerable power, so the power consumption is very high. Different studies have proposed some strategies to reduce power consumption, such as heating the sensitive element in a discontinuous way, which can reduce the power consumption up to 90% [48], but with this method, the accuracy is also reduced and must be considered.

The bottleneck factors impeding the further development of a MOS-based electronic nose are attributed to power consumption, cross-sensitivity (even if recent research has improved the selectivity of solid-state sensors substantially by material doping [49]), and the impact of temperature and humidity [50].

#### 4.1.2. Electrochemical Gas Sensors

An electrochemical gas sensor is generally composed by three electrodes, immersed in an electrolyte solution: the working or sensing electrode (WE), where, depending on the gas, a reduction or oxidation takes place, a counter electrode (CE), where the ions produced by the rection on the WE are deposited, and a reference electrode (RE). Between WE and CE, an electrical current flows that is proportional to the gas concentration. A predefined potential is maintained between WE and RE to guarantee the complete reaction of the target gas. The phenomenon is governed by Nernst law. The generated current is amplified and processed according to the calibration to provide the user a reading in either parts per million or percentage volume. Different materials of electrodes and electrolytes generate electrochemical sensors that have different selectivity and specificity. Cross-sensitivity is not fully eliminated by the choice of a specific sensor composition [46]. However, this phenomenon is limited compared to the cross-sensitivity of MOS sensors and it can be said that they have a high sensitivity and selectivity.

Electrochemical (EC) sensors can be linear or logarithmic. They are small (20 mm), but the conditioning circuit required takes up much space. A possible conditioning circuit is an Analogue Front End (AFE) potentiostat that converts the current signal into a voltage signal and amplifies it. They are characterized by a very low power consumption with respect to MOS sensors due to the low level of electrical current produced. In fact, EC sensors require a strong stage of amplification.

As with MOS sensors, EC sensors are affected by temperature and humidity but at a lower level. The impact can be modelled through the calibration procedure. However, in modern gas sensors, a fourth electrode, the auxiliary electrode (AE), is added. It is not dependent on the target gas and, thus, the current measured at the AE is only influenced by atmospheric condition, mostly by temperature and humidity. In this way, the background current fluctuations of WE can be approximated by the current measured at the AE, as stated by Liang et al. [51].

The limit of EC sensors is their longevity. Their life is influenced by the overall amount of pollution to which they are exposed, but in most cases, they can be reliably used for 2 years. Moreover, they are characterized by a low resilience to extreme weather conditions: a low level of humidity combined with high temperature can dry out the sensor’s electrolyte.

MOS and EC sensors are the most widely used for environmental monitoring, but the choice depends on the goal of the project. If the field of application involves quite stable temperature and weather conditions, EC sensors are recommended because of their specificity and accuracy. Instead, if the application involves a long period of measurement, MOS sensors with a 10-year lifespan are strongly suggested. In terms of calibration, EC and MOS sensors are quite similar with the important difference of how frequently they need to be recalibrated [45].

#### 4.1.3. Non-Dispersive Infrared Sensors

Non-Dispersive Infrared (NDIR) sensors are based on optical transducing mechanisms based on the Infrared Gas Absorption Spectra.

They comprise a gas chamber that is irradiated by IR light. Gas molecules adsorb energy at a narrow absorption band, depending on the specific gas. The Beer–Lambert equation states that the logarithmic relationship between the intensity of the irradiated light and the one measured by the photodetector is directly proportional to the gas concentration. NDIR sensors’ sensitivity and dynamic range depend on the optical path, which is the major design tool for this kind of sensor. For this reason, the dimensions of this kind of sensor can be reduced only to a certain extent because as, they are reduced, the sensitivity worsens [52].

These sensors are very popular for CO_2_ monitoring, among other technologies, even if they are quite expensive.

#### 4.1.4. Photoacoustic Spectroscopy Sensors

The working principle of photoacoustic spectroscopy (PAS) sensors is based on the use of a LED, a photodetector, and a MEMS microphone. The LED is modulated in frequency: when the light hits CO_2_ molecules, they heat up and this causes a translation movement that generates a pressure variation inside the gas chamber. This is sensed by the microphone and produces the photoacoustic signal. After a few milliseconds, the thermal equilibrium is restored and, when the LED is turned off, the CO_2_ cools down, provoking a wave of the opposite sign. If the excitation frequency is near 350 Hz, these two effects constructively interfere, leading to resonance. Calibrating the system with a known CO_2_ concentration allows us to find a coefficient to retrieve the CO_2_ concentration from the photoacoustic signal.

As reported by Scholz et al. [53], a setup that exploits the photoacoustic effect, instead of the typical NDIR setup with an equal optical length, shows an increased sensitivity by a factor 9. Therefore, photoacoustic sensors can reach a miniaturized footprint with an optical path of a few millimeters, performing a reliable evaluation of the CO_2_ concentration. In the field of CO_2_ monitoring, Sensirion and Infineon have recently launched miniaturized CO_2_ sensors, with a reduced dimension and digital output.

### 4.2. Particulate Matter Sensors

There are different techniques to measure the content of particulate matter in the air [54]. These techniques can be divided into two groups: direct continuous measurement and filter-based gravimetric measurement. The latter is the reference method in government agencies. It collects PM over a filter that is periodically weighted in a laboratory; therefore, it is a time- and human-resources-consuming approach, and the time resolution of the measurement is significantly poor. Direct measurement techniques are based on the shift in oscillation frequency of a sensing glass tube or exploit beta-attenuation or the Black Smoke Method, which correlates the darkness of a filter with the PM mass concentration. However, all these sensors are expensive, heavy and have vast dimensions. In the wearable application field, the more widespread approach is based on light scattering. Particulate matter particles enter the measuring chamber and are hit by a laser beam. A photodiode detects the scattered light and, by analyzing the intensity and dimension, it can be evaluated. The amount of detected light by the photodiode reveals the particle count. Then, the mass concentration is assessed by considering the count number, type, and shape of the particles, but it can introduce errors. However, this approach has a short sampling time, in the order of seconds or minutes, and it allows the measurement of different sizes of particles simultaneously. The smallest dimension reached is 41 × 41 × 12 mm^3^ by Sensirion SPS30.

In an article published in 2017 [55], acoustics, electrical sensing and microfluidics were reported as possible future approaches to the PM detection problem, even though the read-out electronics still have problems in the direction of miniaturization. ST Microelectronics have proposed a feasibility study [56] of a piezoelectric PM detector that could easily fit on wearables in the future. This detector is constituted by a circular membrane with a piezoelectric layer on top, placed between two electrodes. The membrane has a lower resonating frequency when particulates land on the piezoelectric layer and so the mass concentration can be retrieved.

In Table 2, a summary of the different sensors, principal pollutants detected and advantages and disadvantages is presented.

## 5. Wearable and Portable Commercial Products for Environmental Monitoring

Several wearable and portable commercial products for environmental monitoring have been proposed and developed detecting the pollutants previously discussed. In this scoping review, some of the most relevant solutions are reported.

TZOA is the most promising wearable commercial products for environmental monitoring [57]. This device measures PM10 and PM2.5 through a self-designed PM sensors exploiting light scattering, atmospheric pressure, humidity, temperature, ultraviolet exposure and ambient light, and a fan is included in the package to prevent the air from stagnating. This wearable air quality tracker cost less than a hundred Euros, and it could be clipped to the clothing because it is compact and lightweight. It made its debut at Wearable World 2015 and was evaluated by Time Magazine as the Best Inventions of 2015. However, after 2016, no more information about the product has been available. Nowadays, TZOA has joined a company based in Vancouver, Haven, focusing on fixed stations for indoor air quality monitoring [58].

ATMO [59] is a company born in 2016 based in San Francisco that operates in the field of environmental sensing. Atmotube PRO, which costs EUR 350 and is a portable solution that, exploiting light scattering, monitors PM1, PM2.5 and PM10 pollutants, such as dust, pollen, soot, and mold, in addition to a wide range of VOCs in real time. PM typical accuracy is ±10 μg/m^3^ in the range of 0–100 μg/m^3^ and ±10% in the range of 100–1000 μg/m^3^. VOC sensors have a typical accuracy of 10% of the measured values within the range of measure, which is 0–60 ppm. The output is an index of air quality that spans from 0 to 100. It also monitors atmospheric pressure, temperature, and humidity in a very compact packaging. Another portable product is Atmotube PLUS (50 euros), which measures pressure, temperature, and humidity, but in what concerns air pollution, it only monitors VOCs with the same sensor of Atmotube PRO.

**Figure 2:**
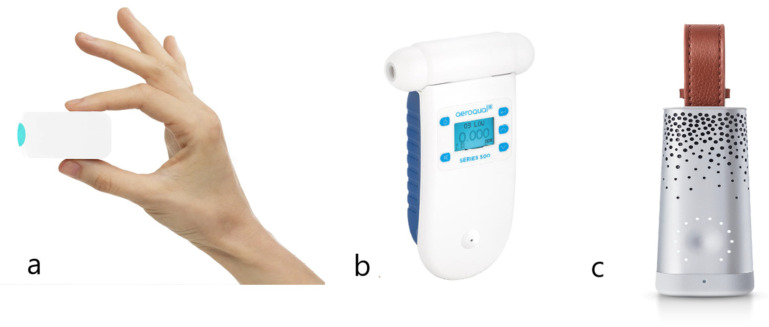
Three commercial products: (**a**) Wynd Air Quality Tracker; (**b**) AEROQUAL’s portable air quality monitor; and (**c**) Plume Labs’ Flow.

The Wynd Air Quality Tracker [60] in Figure 2a is a wearable device produced by a Silicon Valley startup. This device senses airborne particulate matter—including dust, allergens, and industrial pollution—in real time through a self-made light scattering sensor. It is associated with an iOS and Android application to interact with the final user.

AirBeam [61] is a portable device that monitors PM1, PM2.5, PM10, temperature and humidity. In the mobile mode, AirBeam communicates measurements to the Android or iOS device every second via Bluetooth. In the fixed mode, AirBeam communicates measurements directly to the AirCasting website every minute via either the WiFi or cellular network. It is mostly suited to detect outdoor pollution, but it can also be used for indoor monitoring.

AEROQUAL [62] devices include a handheld air quality monitor, shown in Figure 2b. It exploits some sensors of GSS and others. It has a “swappable” sensor head that allows users to measure up to 30 common indoor and outdoor pollutants in real time, removing and replacing sensors in seconds. It is provided with a fan to make the air circulate.

i-BLADES [63] is a portable sensor designed to be used as a smartphone cover. The used sensor is Bosch BME 680 in a smartphone case and is provided with an additional battery to provide additional power to the system. However, it only monitors VOCs, and its reliability has not been fully assessed.

The Plume Labs’ Flow device [64], shown in Figure 2c, offers a portable product that measures PM, NO_2_ and VOCs. It exploits light scattering for PM and MOS sensors for NO_2_ and VOCs, and a general accuracy of 90 to 95% correlation with static reference monitors was reported. A fan is added to assure the real air flow is measured. Neural networks are trained to detect pollution patterns to help to determine levels of PM, NO_2_ and VOCs. An Android or iOS application allows us to monitor different air pollutants and offers a description of the causes and suggestions to lower the pollution level. The application provides as a measurement only the Air Quality Index (AQI), which is connected to the most present air pollutant in that moment. It must be recharged on a fixed station, which allows also re-calibration when needed.

Huma-i [65] is a portable device developed in South Korea that measures CO_2_, VOC, PM1, PM2.5 and PM10 in indoor environments. Input ranges are 400~5000 ppm for CO_2_, 0.000~5000 ppm for VOCs and 0~999 μg/m^3^ for PM1.0/PM2.5/PM10. In addition, it provides information about temperature and humidity, and every parameter can be checked real time through an application that sends notifications of changes in air quality through push messages and provides accurate weather information. It also provides a straightforward indication of AQI.

## 6. Wearables for Air Monitoring in the Scientific Literature

In addition to the wearables that are already available on the market, more are being developed in research settings. Some of the most relevant wearable systems found in the literature for environmental monitoring are presented in this section. Projects were chosen based on the most valuable results or based on the valuable technological solutions that can be useful for the future design of a wearable device.

In the work by Shum et al. [49], a plug-in module for CO monitoring in a wearable system was designed. The bracelet Bracelet SystemF is a flexible device that is organized in modules. This design allows adding or removing modules according to the specific needs. It includes a GPS module for localization and temperature as well as pressure sensors to monitor thermal comfort. As reported by different studies, a paramount problem in designing a wearable system for air monitoring is the accuracy of low-cost sensors. In this research, the calibration was conducted through the comparison to a mass flow controller with CO gas. Electrochemical sensors were compared, among other parameters, regarding sensitivity. Larger sensors show a better sensitivity, which can span from 70 nA/ppm to 200 nA/ppm depending on the manufacturer.

Another example is CitiSense [66] and it monitors CO, NO_2_ and O_3_ by means of electrochemical sensors, in addition to other environmental parameters, i.e., temperature, humidity and barometric pressure. The system, according to the authors, shows enough sensitivity to measure CO, NO_2_ and O_3_ levels down to 1 ppm, 20 ppb and 10 ppb, respectively. It is equipped with a Bluetooth module to communicate with a smartphone. Most of the signal processing is performed by the board, while the smartphone is responsible for storing pollution data and for aggregating them with GPS, location, and timing. The user can check air quality levels on a smartphone app. To test the user experience, two protocols were conducted, and an interview was performed after each protocol was made to collect opinions. The first protocol lasted 2 weeks, participants were cyclists, and the aim was to have feedback about the possibility of continuously monitoring air quality. The second one lasted four weeks, involving eight participants, and the system was equipped with a personal online map that allows the users to access their historical data. Important user feedback from the two protocols for designing an air monitoring system was that, since it is portable and must be attached to a purse or a bag, it is used only when a purse or bag is present, posing a limit to a continuous measurement. The outcomes of the study highlighted that adding to the application the possibility of sharing air quality monitoring through social networks is captivating. This system was used in a European project called Citi-sense that lasted from 2012 to 2016. Computer codes and outcomes are publicly available.

**Figure 3:**
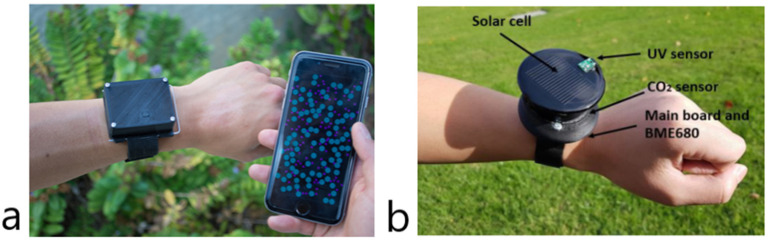
Wrist-worn wearable environmental monitors from the analyzed research papers: (**a**) Mypart; and (**b**) We-safe.

Mypart [67], shown in Figure 3a, is a wrist-worn device to monitor PM. In this case, the sensor for PM detection was designed by the research group to be smaller than the commercially available ones. To allow a good air flow, the system design consists of a channel with an inlet, a fan powered with 3 V and an outlet. A first validation study was conducted by evaluating the Mypart performance against a commercial, expensive handheld device in 20 locations, both indoor and outdoor, to test the different conditions that can be encountered in everyday life. Then, a study of user experience was conducted, consisting in three steps: a first interview, a “walkabout” and a follow-up interview. In the first interview, there was a dialog to understand the extent to which users were informed regarding the pollution problem, and then the subjects were shown how to use the device and first records were collected. The walkabout consisted in a 40 min walk over a 2.7 km distance. The participants were asked to follow a determined route and stop in eight specified locations, chosen to highlight the variability in air pollution. A follow-up interview was made to collect opinions. Someone suggested to attach the system to an object that is used every day. Someone else stated that is useful to change some behaviors, such as cleaning the house or decide where to train. Some participants stated that it can cause frustration, for instance, if someone discovers that their house is in a highly polluted area but has no money to relocate. Another useful aspect of the study is that Mypart is designed to easily adapt to the function of carabiner, a backpack strap, a wristwatch and even be embedded into a toy airplane, thus making it highly flexible in terms of possible applications.

Dieffenderfer et al. [68] present a three-modal system to analyze the effects of pollution on subjects with chronic asthma. It consists of a wristband, a chest patch, and a spirometer. The extracted parameters regarding environment are ozone and VOCs through MOS sensors, temperature, and relative humidity, while, in what concerns physiological parameters, the system detects the heart rate thanks to PPG and ECG; the respiratory rate through PPG; and skin impedance, three-axial acceleration, wheezing via a microphone and expiratory airflow through a spirometer. This system allows the detection of asthma attack onset and permits the study of the relationship between pollution level, activities, and asthma. However, the presented design is not user friendly and cumbersome to use in everyday life.

Mallires et al. [69] developed a wrist-worn device that detects ozone and TVOCs with a MOS sensor, temperature, humidity and activity level (with an accelerometer). The device was developed to monitor air pollution status for people with respiratory problems. Interestingly, the rapidity in response to a rapid change in environment with different pollution levels is tested. The protocol consists of moving the device between indoor and outdoor environments at two-hour intervals for 10 h. The accuracy is reported to be good with an average root-mean-squared error (RMSE) of 5.4 ppb indoor and 9.9 ppb outdoor.

Another example is the work by Cho [70], in which a wearable device that measures O_3_, CO, NO_2_ and VOCs is presented. This wrist-worn prototype includes MOS sensors and accelerometers to track user activity. A sleep mode was used for some sensors if the condition allowed it. The display allows a direct visualization of results. This study, however, also proposed a fixed version for indoor environments that, thanks to proximity sensors, is activated only when a human is detected. 

The device proposed by Fletcher et al. [71] monitors O_3_, SO_2_ and VOCs and is called Eco-Mini by the authors. The gas sensors used are the EC sensors and, even if the sensors can detect changes of few ppb, in practice, electrical noise limits accurate reading in the range of 50–100 ppb. Interestingly, another study included a microphone to consider also acoustic pollution. Accelerometers are used to track activity. To save power, Bluetooth, data logging and GPS can be disactivated by the smartphone, and there is also a button over the case to disable Bluetooth.

Ubiqsense is a wearable device for monitoring NO_2_, CO and SO_2_ with EC sensors, thermal parameters, acoustic noise, and activity (with an accelerometer). The interesting thing is the multi-layer form factor, which allows the use of a considerable number of electronic components but in a reduced space. It is a valuable technological solution for a wearable device. The reported detection range of the NO_2_ sensor is between 0.1 and 75 ppm, but it is not clear if it is a result of the testing phase or is only written in the datasheet of the sensor.

CO and CO_2_ are the gases monitored by We-safe [72], which can also detect temperature, relative humidity (RH) and ultraviolet (UV) light. The CO_2_ sensor is an NDIR sensor with a reported accuracy of ±50 ppm. The system is powered by solar cell and uses LoRa technology to connect to the gateway. It introduces the test of two identical wearable devices to monitor variability between them. The device is shown in Figure 3b.

## 7. Limitations of Wearable Low-Cost Systems

The main limitation of a low-cost wearable system for environmental monitoring is the accuracy of data. It determines the degree of trust in the system. In the scientific literature, it is reported [36] that there are two kinds of errors: internal and external.

Internal errors include dynamic boundaries and systematic errors that can be solved by correcting offset, gain, nonlinear response, and signal drift. Dynamic boundaries are the ranges within which the sensor is sensible. The low detection limit is fundamental. In fact, if concentrations are lower than that, the noise is very high so that the output signal is no longer accurate. Systematic errors are mainly characterized by an offset along all the sensitive range or only at some interval of concentration. External errors are caused by the surrounding environment dependence on temperature and humidity and by low selectivity. An increase in humidity is related to a lower selectivity.

The calibration of environmental sensors should be performed before and after the deployment.

Another limitation is that environmental sensors are either only portable, so not suitable for a person moving, or they are wearable but generally bulky and cumbersome. If wrist-worn devices, such as those in Figure 3, are considered, it must be noted that generally many people already have watches or smartwatches, so another device could be considered an impediment.

## 8. Exposure Estimation Adding Activity Recognition

When switching from walking to running, the inhalation dosage increases by 78% for CO and 28% for PM2.5 and smaller particles, as reported by Singla et al. [25]. This is only an example of how important it is to consider human activity while estimating actual exposure [73].

This leads to the distinction between population exposure and individual exposure, which is related also to the individual activity daily pattern [37]. There is, in fact, an increasing interest in the simultaneous evaluation of environmental parameters and activity parameters, for instance, in the case of running to allow the users to choose the least polluted areas for their training [74].

Starting from the 1980s, researchers tried to attach the activity information in exposure studies. For instance, in 1983–1985 in Denver and Washington, 1200 subjects were recruited and asked to carry with them a pollution monitor and to annotate activities and microenvironments over 24 h [75]. The National Activity Pattern Survey was the first US survey, with the support of EPA, that tried to retrieve general information about human activity to feed into an exposure model. Activities were annotated by the participants, and researchers played particular attention to activities that can vary the inspiration rate, for instance, running, and activities that can produce air pollutants, such as painting. This was only an initial step in the path towards the comprehension of personal exposure.

Monitoring Personal Air Pollution Exposure (PAPE) is one of the major concerns worldwide. Exposure in a certain period called *p*, which accounts for the actual inhaled quantity of the pollutant, can be calculated as:

Exposure(p)=SZ(p)∗VE

where SZ(p) is the aggregated pollution value, measured as:

$$SZ\left(p\right)=\overset{n}{\underset{t=i}{\sum }}\left(Z_{t_{i+1}}+Z_{t_{i}}\right)\left(t_{i+1}-t_{i}\right)$$

where $t_{i+1}-t_{i}$ is lower than 10 min, *Z* represents the concentration of the considered pollutant and *VE* represents minute human ventilation. Minute ventilation is associated with the activity type performed by the subject and is known to increase with increasing activity intensity. *VE* (m^3^/min) has different values according to the performed activities, which are “run”, “walk”, “rest” or “transport”, and “cycle” [76]. The type of activity and position of subjects are detected through Moves, a mobile application. There are several studies combining activity and position, which can be used as a proxy for environmental conditions if environmental data with adequate granularity are available. A study conducted in Barcelona [77] reported the use of a smartphone app, called CalFit, to collect information about the position and activities of the subject.

The great limitation of this kind of approach is that VE variability is not considered. VE has a great inter- and intra-subject variability, as highlighted by the EPA Exposure factor handbook of 2011. A clear example is that of vulnerable subjects, such as asthmatic or subjects with pathologies that affects the respiratory system, but it is also true for healthy subjects. That is why a more accurate detection of the actual VE seems to be a valuable approach to develop the understanding of the effect of pollutants on health. This could be achieved by estimating minute ventilation with wearable devices that are able to detect changing ventilations with activity [78]. There are several examples of commercial and research devices and garments that can acquire a relative minute ventilation signal [79]. 

Projects of this kind can be inserted in a participatory network that develops a participatory sensing, because the public can now objectively record, analyze, and discover a variety of patterns concerning important issues in their lives, such as health, environmental quality, and traffic. It is important to highlight the guidelines in the design of technologies for this purpose. For this reason, devices should be easily accessible, highly usable and at an acceptable cost.

## 9. Indices to Describe Air Quality

Various air quality indices, summarizing concentrations of different pollutants, are used to provide user-friendly information. In fact, mere concentration values can be difficult to be interpreted by non-specialists. The most widely known index to describe environmental data is the Air Quality Index (AQI). However, there is not a standardized way to evaluate AQI. In fact, according to the region or the country, the concentrations of pollutants that determine the AQI scale from “Good” to “Hazardous” are different [36]. Since data through a wearable device can be presented with a higher sample frequency, for instance, of 1-min, EPA proposed a pilot study in 2016 deploying another approach, called Sensor Scale. It provides feedback to the user about O_3_ and PM2.5 in 1-min. To date, no more information dealing with this kind of scale or why not to use it has been found. Probably, the major reason is because there is no evidence about what a 1 min exposure means for health. It is still a good attempt to create a new way to understand and visualize data.

The main drawback of most existing indexes is that they refer to outdoor air quality: people generally spend more time indoors than outdoors, so the required standard must be even more restrictive inside buildings. Recommendations for indoor air are different from those for outdoor air and, in particular, are based on the European Union (2008) directive for Clean Air and the World Health Organization (2010a, 2010b) guidelines for selected pollutants for indoor air quality [31].

To access indoor environmental quality (IEQ), the IEQ index can be used. However, there is not a standardized method to calculate this index. As reported by Coulby et al. [32], there are two main approaches: the first one uses the index to collect environmental factors that are temperature, humidity, CO_2_, VOCs, PM levels, illuminance and sound; the second one evaluates the sample mean of AQI, thermal, visual and acoustic comfort indexes. The non-univocal definition of the IEQ-Index makes it difficult to compare different studies. The objective evaluation of indoor air quality remains an open research question.

## 10. Possible Applications

Devices for air quality detection can be helpful to assess health impact, as stated by Dias and Tchepel [37]. Environmental monitoring is deemed essential in the field of telemonitoring of patients with resoiratory problems [57] and it has been used in home-based telemedicine platforms [80]; adding a wearable to a platform would be the natural evolution of the previously mentioned applications.

It can be used to suggest the best path according to pollution, to go from point A to point B by taking the route with lower levels of pollution [81]. For instance, it can be useful for people with asthma to avoid heavy pollution or pollen area or for teachers to plan which park to go with students. There are already different studies that consider air pollution in the routing algorithm, for instance, by minimizing PM2.5 [82] or a combination of parameters [83]. In the latter study, the best trip is chosen by considering a cost function that includes two factors, distance, and pollution, with different weights, which can be adjusted by the user.

To this aim, the forecasting of air pollution is necessary. In forecasting, particular attention on weather condition must be paid, as pointed out by multiple studies [84,85].

Furthermore, using wearables for environmental monitoring will have an impact in develop personal awareness. It can enhance changes in personal habits both outdoors and indoors [86]. In the study by Boso et al., one of the collected opinions was that participants were more confident in what the personal instrument said more than on information on newspapers or television.

Additionally, knowledge of environmental conditions can support collective intervention and pollution control policies [37]. A case study of air quality in two oncology units in the European Union [87] consisted in monitoring of TVOCs, PM2.5 and CO_2_ for several months, and showed a considerable impact of human presence, usage of disinfectants, cleaning and pharmaceutical products on the weekday pattern of pollution. This information can help in ventilation strategies, scheduling and even to an improved design of waiting rooms. It would be interesting to approach pollutant exposure as the dosimetry does for ionizing and non-ionizing radiation. For instance, specialized personnel exposed to radiation constantly wear a dosimeter to avoid an absorbed dose that can cause impairments. 

Another case study dealt with air monitoring in schools [88]. After detecting that two-thirds over 100 schools in Switzerland exceeded the CO_2_ limit value of 2000 ppm, an intervention of strategic ventilation was undertaken for 25 of them. The results showed that the mean CO_2_ levels decreased from 1600 ppm to 1097 ppm and the proportion of teaching time in the 400–1400 ppm range increased from 40% to 70%. These great outcomes have been reached while maintaining ventilation times, such as the one with natural ventilation. This confirms the huge impact of knowing pollution levels and implement easy solutions.

Finally, in the field of early childhood education and care centers [89], a study highlighted that PM2.5 levels had the highest value in the period when children conduct physical activities outdoors. The possible damage would be particularly severe because exposure occurs in a period of rapid lung development. The knowledge of the pollution level over a region can be a mean to design ECEC centers in lower polluted areas.

## 11. Conclusions

This scoping review focused on wearable devices for environmental monitoring. First, the main pollutants were presented, followed by sensing technologies that are used for the parameters of interest. Selected examples of wearables and portables were divided into commercially available and research-level projects. While many commercial products are in fact portable, there is an increasing interest in using a completely wearable technology. This allowed us to correlate the pollution level to other personal information (performed activity, position, and respiratory parameters) and thus to estimate personal exposure to given pollutions. The fact that there are no univocal indices to estimate outdoor or indoor air quality is also an open problem. Finally, applications of wearables for environmental monitoring were discussed. Combining environmental monitoring with other devices would allow people to better choose where to perform sports activities, especially in highly polluted areas, and provide detailed information on the living conditions of individuals.

## Figures and Tables

**Table 1:** Summary table of the major pollutants, their sources and their impact on human health.

Pollutant	Sources	Health Impact
PM10 PM2.5 Ultrafine PM	Resuspension of soil of industrial dust Coal or oil combustion Diesel engines (90% of PM2.5 emissions) [6] Transformation products of NO_x_, SO_2_ and organics Anthropogenic	Premature death if heart or lung diseases are present, nonfatal heart attacks and irregular heartbeat Asthma, decreased lung function and increased respiratory symptoms, such as irritation of the airways, coughing or difficulty breathing Hypertension [7] Depressive symptoms [8]
NO_2_	Photochemical reaction of NO with O_3_	Aggravates respiratory symptoms, especially for children, older adults, and asthmatics
O_3_	Formed via UV (sunlight) and pressure of other key pollutants	Worsening of bronchitis, emphysema, and asthma Reduction in lung function and inflammation of the lining of the lungs Long exposure: can permanently damage lung tissue
SO_2_	Combustion of coal or oil Factories pertaining to chemicals, paper, or fuel	Asthmatics are the sensitive category (coughing, wheezing, and chest tightness) Long exposure affects everybody
CO_2_	Anthropogenic Deforestation and the burning of fossil fuels, such as coal, oil, and natural gas	Low: dizziness and headaches High: unconsciousness and dyspnea
VOCs	Fuel combustion, gasoline evaporation or solvents Cooking Floor surface materials (PVC/vinyl, linoleum) [9]	All effects related to O_3_ (VOC involved in O_3_ formation) Some toxic per se (e.g., causing cancer)
CO	Engines burning fossil fuels Emitted from vehicles, furnaces, and heaters	Sensation of dizziness, fatigue, and headache Dangerous for people with heart disease

**Table 2:** Summary table of the major sensors’ technologies, principal detected pollutants, advantages, and disadvantages.

Sensor	Principal Detected Pollutant	Advantages	Disadvantages
MOS	CO, CO_2_, NO, NO_2_, NO_x_, O_3_, NMHCs	Small size, low prize, high sensitivity, wide input range, high resilience to environmental condition, longevity	Power consumption, cross-sensitivity, recalibration, strong impact of temperature and humidity
EC	CO, NO, NO_2_, NO_x_, O_3_	Low power consumption, specificity, accuracy	Longevity, resilience, strong amplification, dimensions
NDIR	CO_2_	Sensitivity, reliability	Dimensions, cost
PAS	CO_2_	Dimensions, cost, sensitivity, reliability	Not reported
Light scattering	PM	Response time, accuracy	Dimensions, cost

## Data Availability

Not applicable.
